# Imaging Voltage with Microbial Rhodopsins

**DOI:** 10.3389/fmolb.2021.738829

**Published:** 2021-08-25

**Authors:** Xiao Min Zhang, Tatsushi Yokoyama, Masayuki Sakamoto

**Affiliations:** ^1^ Department of Pathophysiology, Guangdong Provincial Key Laboratory of Brain Function and Disease, Zhongshan School of Medicine, Sun Yat-sen University, Guangzhou, China; ^2^ Department of Optical Neural and Molecular Physiology, Graduate School of Biostudies, Kyoto University, Kyoto, Japan; ^3^ Precursory Research for Embryonic Science and Technology (PRESTO), Japan Science and Technology Agency, Kyoto, Japan

**Keywords:** voltage imaging, microbial rhodopsins, photocycle, FRET (förster resonance energy transfer), optogenetics, *in vivo* imaging

## Abstract

Membrane potential is the critical parameter that reflects the excitability of a neuron, and it is usually measured by electrophysiological recordings with electrodes. However, this is an invasive approach that is constrained by the problems of lacking spatial resolution and genetic specificity. Recently, the development of a variety of fluorescent probes has made it possible to measure the activity of individual cells with high spatiotemporal resolution. The adaptation of this technique to image electrical activity in neurons has become an informative method to study neural circuits. Genetically encoded voltage indicators (GEVIs) can be used with superior performance to accurately target specific genetic populations and reveal neuronal dynamics on a millisecond scale. Microbial rhodopsins are commonly used as optogenetic actuators to manipulate neuronal activities and to explore the circuit mechanisms of brain function, but they also can be used as fluorescent voltage indicators. In this review, we summarize recent advances in the design and the application of rhodopsin-based GEVIs.

## Introduction

Probing functional neural circuits at high spatiotemporal resolution is crucial for understanding how neuronal populations work together to generate behavior. To do this, it is necessary to measure neural activity from multiple neurons simultaneously. Electrophysiological approaches are used to measure membrane potential as the gold standard. However, the results acquired by recording with electrodes lack spatial resolution and genetic specificity. Optical imaging with genetically encoded indicators can overcome these drawbacks and monitor the activity of large numbers of neurons simultaneously.

Since somatic calcium influx is coupled with action potentials (APs), the activity of large numbers of neurons can be monitored simultaneously using calcium imaging as an indirect measurement of neuronal firing with an excellent signal-to-noise ratio (SNR) (Yuste and Katz, 1991; Grienberger and Konnerth, 2012). Genetically encoded calcium indicators (GECIs) are the most widely used to monitor neural activity *in vitro* and *in vivo* (Nakai et al., 2001; Tian et al., 2009; Zhao et al., 2011; Akerboom et al., 2012; Ohkura et al., 2012; Chen et al., 2013; Inoue et al., 2015, 2019; Dana et al., 2019). With calcium imaging, it is possible to measure spiking activity from thousands of neurons in neural circuits with single-cell resolution in behaving animals (Ziv et al., 2013; Sofroniew et al., 2016; Stirman et al., 2016; Ota et al., 2021). Furthermore, in addition to measuring the activity in the somata, the activity in other subcellular domains like dendritic spines and axonal boutons can be measured *in vivo* (Chen et al., 2013; Broussard et al., 2018; Inoue et al., 2019).

However, calcium dynamics revealed by fluorescent calcium indicators are not a direct measurement of membrane potential. Thus, calcium imaging is limited in its ability to provide a complete description of neuronal activity. First, somatic calcium imaging readouts only APs (Smetters et al., 1999). Subthreshold excitatory or inhibitory synaptic inputs are practically invisible in somatic calcium signals, making it difficult to monitor the relationship between the synaptic inputs and outputs. Second, due to biophysical constraints, calcium dynamics are significantly slower than the timescale of membrane potential dynamics. Therefore, when neurons fire a burst of spikes at > 40 Hz, it is difficult to assess the number of spikes and spike timimgs quantitatively with population calcium imaging (Smetters et al., 1999). Third, calcium dynamics are shaped by complicated interactions between ionic diffusion and extrusion, and they can be significantly altered by intrinsic and extrinsic calcium buffers and the expression of calcium indicators themselves (Neher, 1998). Calcium imaging is not an ideal method to measure neural activity for these reasons.

Voltage imaging, on the other hand, can directly monitor the electrical activity of each neuron, including subthreshold events (Peterka et al., 2011; Storace et al., 2016). Intensive efforts have been made to develop genetically encoded voltage indicators (GEVIs) (Akemann et al., 2010; Jin et al., 2012; Tsutsui et al., 2013; St-Pierre et al., 2014; Piao et al., 2015; Inagaki et al., 2017). These genetic indicators can target and measure specific cell types or subcellular compartments (Kwon et al., 2017). Newer GEVIs can detect subthreshold activity that is not detectable with calcium imaging both *in vitro* and *in vivo* (Bando et al., 2019; Villette et al., 2019), making it possible to generate more accurate decoding of brain functions. Therefore, voltage imaging using GEVIs appears to be a powerful tool that can supersede calcium imaging.

Microbial rhodopsins were initially used for optogenetic control of membrane potential (Boyden et al., 2005; Han and Boyden, 2007; Chow et al., 2010). It turned out that these rhodopsins also show a membrane voltage-dependent fluorescent change that is derived from the retinal chromophore (Kralj et al., 2011a, 2011b; Kojima et al., 2020). Compared with other types of GEVIs (ion channel-based or voltage-sensitive domain (VSD)-based), rhodopsin-based GEVIs display higher sensitivity and faster kinetics, and the use of this type of sensor has become widespread (Flytzanis et al., 2014; Hochbaum et al., 2014; Piatkevich et al., 2018; Adam et al., 2019; Chien et al., 2021). In this review, we will introduce recent advances in the design and the application of rhodopsin-based GEVIs. We hope that this review will enable the readers to choose the optimal GEVIs for their specific application and inspire the development and improvements of GEVIs.

### Mechanism of Microbial Rhodopsins as a Voltage Indicator

Due to the low quantum yield of the retinal chromophore, little attention has been given to the fluorescence of rhodopsin. Kralj and colleagues developed a new type of GEVI based on microbial rhodopsins and their fluorescence. They found that proteorhodopsin, a light-driven proton pump discovered from uncultivated marine γ-proteobacteria, can detect the electrical activity in bacteria (Kralj et al., 2011b). By exploiting its properties, they developed a proteorhodopsin optical proton sensor (PROPS), and the authors measured membrane voltage fluctuations in E. Coli (Kralj et al., 2011b). However, PROPS does not localize to the plasma membranes of eukaryotic cells efficiently. They further screened other microbial rhodopsins and found Archaerhodopsin-3 (Arch) from *Halorubrum sodomense* reliably expressed and trafficked to the plasma membrane well in mammalian and successfully reported membrane potentials in neurons (Kralj et al., 2011a).

**FIGURE 1:**
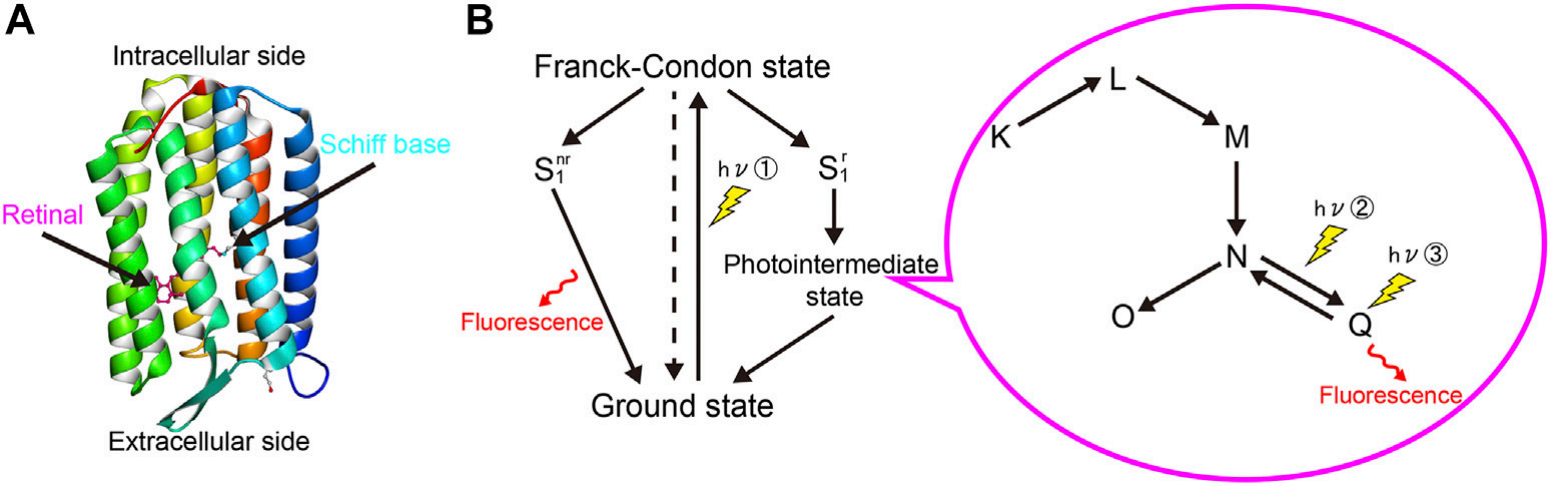
Fluorescence mechanism of microbial rhodopsins. **(A)** Crystal structure of Arch (PDB code: 6GUZ). Rhodopsin is a membrane protein with a seven-fold transmembrane alpha-helix structure and consists of a protein moiety called opsin and a retinal chromophore that is covalently bound to the apoprotein via a Schiff base. **(B)** Photoreaction scheme of microbial rhodopsins. The spontaneous emission (left) occurs from the nonreactive S_1_ state. The photointermediate fluorescence (right) is from the Q-intermediate state produced by a photon absorption of the N-intermediate in its photocycle. Photointermediate fluorescence arises through the sequential action of three photons (① - ③). The dashed line represents the non-radiative relaxation process.

Arch serves as a light-driven outward proton pump, and it is utilized as an inhibitory optogenetic actuator that is activated by green light (Chow et al., 2010). Retinal is bound to a specific lysine residue (K226) in the seventh helix of apoprotein (opsin) via a Schiff base (Figure 1A). When absorbing light, rhodopsin molecules in the ground state lead to the Frank-Condon state and then form the reactive state (S_1_^r^) or nonreactive S_1_ states (S_1_^nr^) within several tens of femtoseconds (Figure 1B). When the rhodopsin molecule in S_1_^nr^ is illuminated, the excess energy is released as fluorescence, and the molecule returns to the ground state. This spontaneous emission is a common property of microbial rhodopsins (Figure 1B) (Nakamura et al., 2008; Kojima et al., 2020). In the reactive state (S_1_^r^), on the other hand, the retinal is isomerized from the all-*trans* to the 13-*cis* form. This light-induced isomerization triggers further distinctive photointermediates such as the K-, the L-, the M-, the N-, and the O-states, followed by returning to the ground state (Figure 1B). It also forms a Q-intermediate state when absorbing light in the N-intermediate state (Figure 1B) (Ohtani et al., 1992; Maclaurin et al., 2013). The Q-intermediate state emits fluorescence that is about 100 times larger than that of spontaneous emission. By comparing these different fluorescence intensities in mammalian neurons, rhodopsin fluorescence in Arch was derived from the Q-intermediate state (Kojima et al., 2020). Also, such photointermediate fluorescence arises from a sequential three-photon process. Photon 1 initiates the photocycle that Schiff base is protonated, and Arch transits from the ground state to N-intermediate state. Photon 2 further generates a Q-intermediate state, and photon 3 enables yield fluorescence (Figure 1B) (Maclaurin et al., 2013).

### Microbial Rhodopsin-Based Voltage Indicators

**FIGURE 2:**
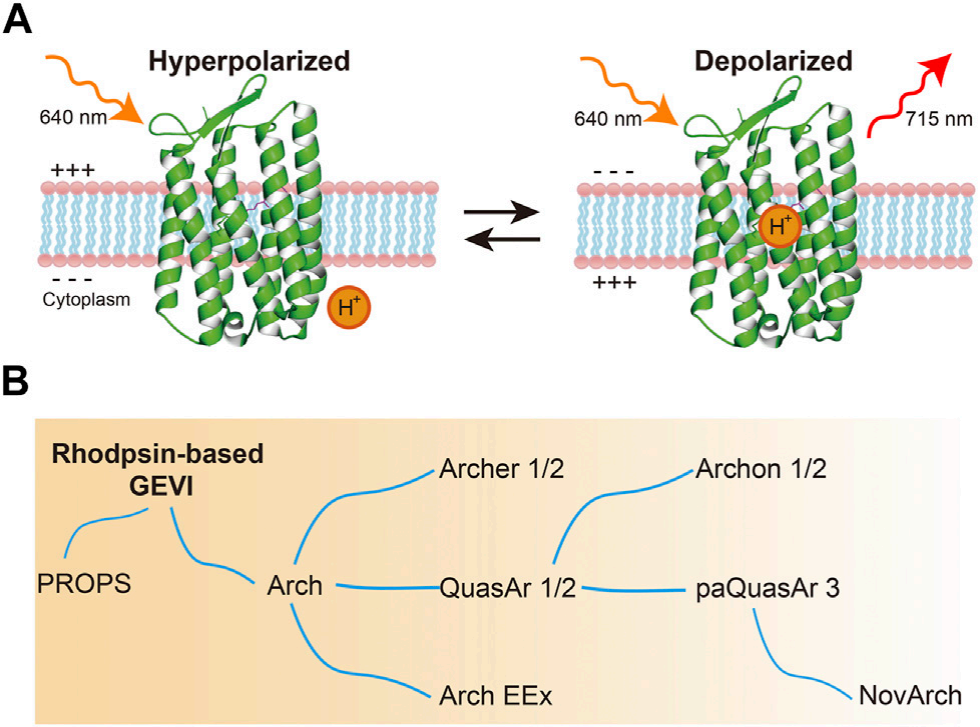
Microbial rhodopsin-based GEVIs. **(A)** Voltage sensing mechanism of microbial rhodopsin-based GEVIs. Rhodopsin-based GEVIs report voltage changes through the fluorescence intensity changes of retinal chromophore caused by protonation of the Schiff base in the photointermediate state, not in the ground state. **(B)** Evolution of microbial rhodopsin-based GEVIs.

Arch can effectively reflect membrane potentials with extremely high temporal resolution. For voltage imaging, Arch and its mutants are excited by red light (640 nm) and emit in the infrared wavelength (peak at 715 nm) (Figure 2A) (Kralj et al., 2011a). Such voltage sensitivity arises through protonation of the Schiff in the photointermediate state, not the ground state. However, their practical applications are limited by their weak fluorescence (equal to 1/500–1/50 of EGFP) and insufficient SNR (Kralj et al., 2011a; Maclaurin et al., 2013). To overcome these problems, efforts were devoted to improving Arch performance (Figure 2B, Table 1). Mutating residues related to the photocycle or around the retinal Schiff base could significantly modify Arch’s brightness and SNR. Consequently, several GEVIs available *in vitro* and *in vivo* were developed (Kralj et al., 2011a; Gong et al., 2013; Flytzanis et al., 2014; Hochbaum et al., 2014; Piatkevich et al., 2018; Adam et al., 2019).

**TABLE 1:** Comparative performance of rhodopsin-based genetically encoded voltage indicators.

GEVI	Rhodopsin	Fluorophore	ΔF/F (%)	τ_on_			τ_off_			References
				τ_1_ (ms)	τ_2_ (ms)	%τ_1_	τ_1_ (ms)	τ_2_ (ms)	%τ_1_	
**Microbial rhodopsin-based GEVIs**										
Arch	Arch	Retinal	40	0.6	—	—	0.25	1.9	67	Kralj et al. (2011a)
Arch (D95N)	Arch	Retinal	60	41	—	—	—	—	—	Kralj et al. (2011a)
Archer1	Arch	Retinal	85	—	—	—	—	—	—	Flytzanis et al. (2014)
Archer2	Arch	Retinal	60	—	—	—	—	—	—	Flytzanis et al. (2014)
QuasAr1	Arch	Retinal	32	0.053 ± 0.002	3.2	94	0.07	1.9	88	Hochbaum et al. (2014)
QuasAr2	Arch	Retinal	90	1.2 ± 0.1	11.8 ± 1.5	68	1	15.9	80	Hochbaum et al. (2014)
Archon1	Arch	Retinal	43	0.06 ± 0.06	8.1 ± 0.5	88	1.1 ± 0.2	13 ± 3	88	Piatkevich et al. (2018)
Archon2	Arch	Retinal	19	0.06 ± 0.01	6.7 ± 0.4	70	0.17 ± 0.01	7.0 ± 0.5	92	Piatkevich et al. (2018)
QuasAr3_Blue off_	Arch	Retinal	50	1.2 ± 0.2	10.0 ± 1.8	77 ± 5	0.9 ± 0.1	9.0 ± 1.2	91 ± 5	Adam et al. (2019)
paQuasAr3_Blue_	Arch	Retinal	50	0.8 ± 0.04	19.3 ± 1.1	54 ± 2	0.69 ± 0.04	15.8 ± 1.9	69 ± 2	Adam et al. (2019)
SomArchon	Arch	Retinal	30	—	—		—	—		Piatkevich et al. (2019)
**eFRET-based GEVI**										
QuasAr2-Citrine	Arch	Citrine	-13.1	4.8	21	38	3.1	21	62	Zou et al. (2014)
MacQ-mCitrine	Mac	mCitrine	-20	2.8 ± 0.2	71 ± 3	74 ± 2	5.4 ± 0.3	67 ± 11	77 ± 2	Gong et al. (2014)
Ace2N-mNeon	Ace2	mNeonGreen	-18	0.37 ± 0.08	5.5 ± 1.4	58 ± 5	0.50 ± 0.09	5.9 ± 0.9	60 ± 6	Gong et al. (2015)
Ace2N-mScarlet	Ace2	mScarlet	-15	0.79 ± 0.18	2.4 ± 0.6	79.4	1.1 ± 0.32	8.6 ± 2.8	58	Beck and Gong (2019)
VARNAM	Ace2	mRuby3	-10	0.88 ± 0.13	5.2 ± 0.5	—	0.80 ± 0.44	4.7 ± 0.3	—	Kannan et al. (2018)
Voltron_525_	Ace2	JF525	-23	0.64 ± 0.09	4.1 ± 0.6	61 ± 4	0.78 ± 0.12	3.9 ± 0.2	55 ± 7	Abdelfattah et al. (2019)
Positron	Ace2	JF525	18	0.63 ± 0.08	19 ± 6	85 ± 6	0.64 ± 0.10	37 ± 4	90 ± 2	Abdelfattah et al. (2020)
HVI-Cy3	Ace2	Cy3	−39	—	—	—	—	—	—	Liu et al. (2021)
HVI-Cy5	Ace2	Cy5	−20	—	—	—	—	—	—	Liu et al. (2021)

Note: Characterizations were recorded in HEK cells at 22°C. Parameters labeled with colors were recorded in other conditions as following.

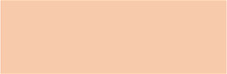
Recorded in HEK cells at 34°C.

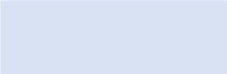
Recorded in neuronal culture at 22°C.

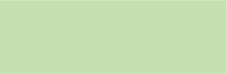
Recorded in neuronal culture at 32°C.

Wild-type Arch generates hyperpolarizing photocurrents upon exposure to an imaging laser. By changing the residue of the first position of proton translocation in the photocycle (D95), the photocurrent could be significantly eliminated; however, this mutation also made the rise time of Arch ∼45 ms slower (Table 1) (Kralj et al., 2011a; Gong et al., 2013). D106 is the primary conduit for protons to protonate and deprotonate the voltage-sensitive Schiff base during modulations of membrane voltage. Gong and colleagues combined the mutation D95N/Q with D106E and generated the GEVIs called Arch-EEN and Arch-EEQ, and these showed a faster response (∼5–15 ms rise time) to APs when compared to Arch-D95N (Gong et al., 2013). Archer1 and 2 were generated based on the spectral shifts mutation of D95E, T99C, and A225M that was reported in *Gloeobacter violaceus* rhodopsin (GR), resulting in higher brightness and SNR (Engqvist et al., 2015). Archer1 also worked as a bi-functional tool, detecting membrane potential with red light illumination and inhibiting neural activity with green light illumination (Flytzanis et al., 2014). Furthermore, Venkatachalam and colleagues developed methods for light-gated photochemical voltage recording by modulating the photophysical properties of Arch. By illuminating a neural circuit during a user-defined “write” interval, a photochemical imprint was formed within each cell of the amount of electrical activity during the write interval. This fluorescence can be probed later (Venkatachalam et al., 2014).

Random mutagenesis was another widely used approach to optimize Arch-based voltage indicators, which significantly advanced their kinetics and fluorescence (Table 1). QuasAr1 contains the mutation of P60S, T80S, D95H, D106H, and F161V, and QuasAr2 was generated by changing H95Q in the QuasAr1. Both of these indicators were characterized by significant improvements in brightness and sensitivity. Notably, QuasAr2 showed an approximately 90% ΔF/F to 100 mV membrane voltage change in HEK293T cells and resolved APs in organotypic slice culture (Hochbaum et al., 2014). A newly developed QuasAr3 (K171R to QuasAr2) further improved the expression level of the indicator and had an excellent membrane trafficking property, allowing one to detecting voltage dynamics *in vivo* (Adam et al., 2019). A point mutation (V59A) in QuasAr3 enhanced the population of the fluorescent from the Q-intermediate state. Thus, QuasAr3 (V59A) resulted in a “photoactivated QuasAr3” (paQuasAr3) that enhanced the baseline fluorescence 2-3-fold upon blue light illumination in HEK293T cells (Adam et al., 2019). NovArch, which introduced mutations of V209I and I213T to paQuasAr3, emits enhanced infrared fluorescence with additional weak two-photon catalytically excitation with any light other than blue light (Chien et al., 2021). Simultaneous one-photon and two-photon excitation of NovArch resolved single cells in an acute brain slice at depth up to ∼220 μm where conventional one-photon excitation wide-field and confocal approaches could not. Additionally, NovArch was able to detect back-propagating APs of dendrites in acute brain slices (Chien et al., 2021). Meanwhile, Piatkevich and colleagues developed a computer-vision-guided high-throughput screening system to optimize GEVI’s brightness and membrane localization. By screening QuasAr2 mutant libraries that were generated by error-prone PCR and site-directed mutagenesis, the authors identified multiple residues of QuasAr2 that yield better performance in brightness, membrane localization, and voltage sensitivity (Piatkevich et al., 2018). Compared to QuasAr2, Archon1 has the additional mutations of T20S, G41A, V44E, S80P, D88N, A137T, T184I, L199I, and G242Q. Archon2 has the additional mutations of T56P, S80P, T100C, T118I, T184I, L199I, and A226C. Archon1 is more resistant to photobleaching, retaining ∼95% of its baseline fluorescence after exposure to intensive light (800 mW/mm^2^) for 15 min, while other Arch-based indicators lost at least 25% fluorescence (Piatkevich et al., 2018). Archon1 had a high performance in its ability to detect neural activity in mouse brain slices (Piatkevich et al., 2018). Compared to Archon1, Archon2 had faster kinetics but had lower sensitivity. Also, the targeting of Archon1 to the soma (SomArchon) by adding a trafficking motif from the Kv2.1 potassium channel improved its SNR and sensitivity (Piatkevich et al., 2019).

Arch-based voltage indicators can be applied in conjunction with spectrally orthogonal optogenetic actuators. This simultaneous stimulation and the corresponding readout of membrane potential via light is called “all-optical electrophysiology” or “Optopatch.” Hochbaum and colleagues generated a blue-shifted channelrhodopsin actuator (CheRiff) to use in combination with QuasAr2. Notably, intense stimulation of QuasAr2 with a red laser did not induce any currents when this new actuator was used in cultured neurons and brain slices (Hochbaum et al., 2014). Later this combination was successfully applied for high-throughput screening of a Na_v_1.7-specific blocker from a library of candidates (Zhang et al., 2016). Moreover, the Cre-dependent transgenic mouse line “Floxopatch,” which expresses the combination of QuasAr2 and CheRiff, enabled the characterization of neural activity in genetically specified cell types in intact tissue (Lou et al., 2016). Another combination of using high-photocurrent channelrhodopsin from *Chloromonas oogama* (CoChR) and Archon1/2 also showed excellent performance (Piatkevich et al., 2018). This strategy has been applied successfully *in vivo* (Piatkevich et al., 2019; Fan et al., 2020).

In addition to optogenetic actuators, these microbial rhodopsin-based GEVIs work together with other sensors, such as calcium indicators and pH sensors. For example, a study imaged the changes in voltage with QuasAr2 and the changes in calcium with GECIs simultaneously to explore the correlations between the voltage variations and APs in neurons (Fan et al., 2018). A similar approach was used in a cardiology study to screen for the protective effect of cardioprotective drug candidates by tracking calcium, membrane voltage, and motion path in human induced pluripotent stem cell-derived cardiomyocytes (Nguyen et al., 2019). In addition, Werley and colleagues developed a technique called MOSAIC (Multiplexed Optical Sensors in Arrayed Islands of Cells) that introduced GEVIs and ∼20 other sensors to various kinds of cultured cells. These multiplexed data collected from multiple recordings allow further exploration of complex physiological responses in multiple cell types (Werley et al., 2020).

### eFRET-Based Voltage Indicators

**FIGURE 3:**
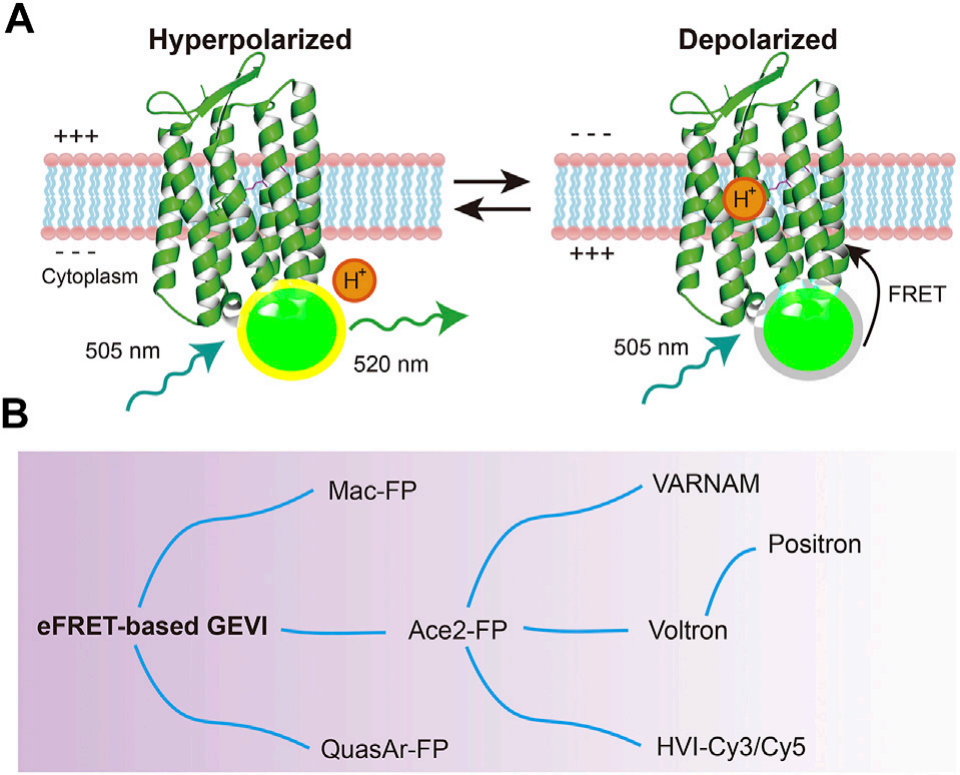
eFRET-based GEVIs. **(A)** Voltage sensing mechanism of eFRET-based GEVIs (Ace2N-mNeon). At a depolarized stage, the Schiff base of microbial rhodopsin is protonated, and the absorbance of rhodopsin changes. This absorption quenches the fluorescence of the appended fluorescent proteins or other bright fluorophores. **(B)** Evolution of eFRET-based GEVIs.

Despite the intensive engineering of Arch variants, the brightness is still lower than that of widely-used fluorescent proteins. To overcome the low fluorescent limitation of rhodopsins, an eFRET (electrochromic Förster resonance energy transfer) strategy was wisely developed. Microbial rhodopsins have absorption spectrum that overlaps with the emission spectrum of widely-used fluorescent proteins. Therefore, fluorescent proteins and other chemical fluorophores can serve as FRET donors, while rhodopsin molecules can serve as FRET acceptors (Bayraktar et al., 2012). eFRET sensors measure the absorption change of rhodopsin through the quenching of an attached fluorescent protein. When neurons depolarize, the fluorescent protein intensity is decreased by FRET from the fluorescent protein to the rhodopsin (Figure 3A) (Gong et al., 2014, 2015; Zou et al., 2014). Thus, these FRET-opsin sensors detect voltage depolarization by the decrease in emission intensity from the fluorescence donor. The rhodopsins utilized in this type of GEVIs were not limited to Arch (Zou et al., 2014). Mac (bacteriorhodopsin from *Leptosphaeria maculans*) (Gong et al., 2014) and Ace2 (bacteriorhodopsin from *Acetabularia acetabulum*) (Gong et al., 2015; Kannan et al., 2018; Beck and Gong, 2019) were also successfully used to generate new indicators that can detect spikes in neurons with fast kinetics and high SNR (Figure 3B, Table1). Microbial rhodopsins have a broad absorption spectrum (Kojima et al., 2020), so various fluorescent proteins with different colors can be utilized as donors (Gong et al., 2014, 2015; Zou et al., 2014; Kannan et al., 2018; Beck and Gong, 2019).

Synthetic fluorescent dyes are also available as FRET donors (Table 1). Voltron has a self-labelling tag domain (HaloTag, 34 kDa) to use Janelia Fluor dyes as the FRET donor (Abdelfattah et al., 2019). The synthetic dyes are more photostable and brighter than the fluorescent proteins, allowing for *in vivo* voltage imaging from large fields of view. Since the absorbance of rhodopsins increases in response to depolarization, eFRET-based GEVIs have negatively sloped fluorescence-voltage relationships. This means that the indicators become dimmer when the neurons depolarize (negative-going). Positron is a positive-going eFRET-based GEVI generated from Voltron, and it possesses identical kinetics and sensitivity as Voltron (Abdelfattah et al., 2020). Furthermore, Arch- and Mac-based positive-going eFRET GEVIs have been developed by modifying the natural proton transport pathway within microbial rhodopsins (Abdelfattah et al., 2020). In addition, other hybrid eFRET indicators, HVI-Cy3 and HVI-Cy5, were reported (Liu et al., 2021). In these constructs, the fluorophore was directly linked to a small peptide (1.6 kDa) inserted at the extracellular loop of the rhodopsin, resulting in high FRET efficiency.

### *In vivo* Voltage Imaging with Rhodopsin-Based Indicators

The primary goal of voltage imaging is to visualize neuronal activity *in vivo.* The development of new probes and an imaging apparatus has shed light on the activity of neurons in behaving animals. The endoplasmic reticulum (ER) and the Golgi export trafficking signal (TS) could significantly improve membrane localization of microbial rhodopsin (Gradinaru et al., 2010). Moreover, a trafficking motif from the soma-localized K_v_2.1 potassium channel could confine the GEVI expression to the soma (Baker et al., 2016), which dramatically decreased the background noise and further improve SNR in the living mammalian brain (Adam et al., 2019; Piatkevich et al., 2019). Combined with these motifs, Arch-based SomArchon and paQuasAr3-s successfully target somata and detect their fluorescence with cellular resolution *in vivo* (Adam et al., 2019; Piatkevich et al., 2019). These indicators also could record neuronal activity from multiple neurons simultaneously in the hippocampus, enabling the study of correlation and coherence of subthreshold activity between pairs of neurons (Adam et al., 2019; Piatkevich et al., 2019). Furthermore, paQuasAr3 enabled the detection of back-propagating APs from dendrites in the hippocampal CA1 region.

MacQ-mCitrine was the first indicator used to investigate neuron membrane voltage of mice and flies *in vivo* due to its bright fluorescence baseline as an eFRET based voltage indictor (Gong et al., 2014). Then, Ace2N-mNeon responded 5–6 times faster than MacQ-mCitrine and provided exquisite spike-timing accuracy. Moreover, Ace2N-mNeon and Voltron were used to measure spiking activity with precise orientation selectivity in the primary visual cortex during the presentation of drifting grating stimuli (Gong et al., 2015; Abdelfattah et al., 2019). Voltron, in particular, showed superior photostability and allowed for over 15 min of continuous imaging (Abdelfattah et al., 2019).

Voltage imaging is possible in freely moving animals using optical fibers. Mashall and colleagues developed a method named “*trans*-membrane electrical measurement performed optically (TEMPO)” to record the changes in the voltage dynamics by using fluctuations in the fluorescence of Mac-mCitrine or Ace2N-mNeon (Marshall et al., 2016). They succeeded in measuring the activity of D1-or D2-dopamine receptor-expressing striatal medium spiny neurons (Marshall et al., 2016). Also, using both an eFRET-based red indicator (VARNAM) in conjunction with TEMPO was able to accurately detect theta (6–10 Hz) and delta (0.5–4 Hz) oscillatory waves in the CA1 region of the hippocampus (Kannan et al., 2018).

The fluorescent fluctuations of rhodopsin-based GEVIs are barely detectable by two-photon microscopy, so the imaging is still restricted to conventional one-photon microscopy that lacks optical sections. To improve the imaging acquisition conditions, Adam and colleagues introduced a digital mirror device for targeted illumination and succeeded in improving the SNR (Adam et al., 2019). Further improvement of SNR was achieved by the use of a spatial light modulator to restrict the illumination area more precisely (Fan et al., 2020). In addition to the performance of GEVIs, optimization of the technology to analyze the data is also essential. Recently, two analysis pipelines (VolPy and SGPMD-NMF) were developed for the processing of voltage imaging data (Cai et al., 2021; Xie et al., 2021). Both pipelines could correct motion artifacts, denoise voltage signals, and extract APs and subthreshold signals from the raw imaging data recorded in mouse and zebrafish brains *in vivo.*

## Conclusion

The development of microbial rhodopsin-based GEVIs helped significantly advance our ability to detect neuronal activity with high spatiotemporal resolution. These indicators bring hope for us to elucidate better how networks of synaptic connections in the brain work together precisely. Furthermore, all living cells have membrane potentials. Therefore, it is interesting to apply voltage imaging to cells other than neurons to elucidate biological phenomena.

However, the rhodopsin-based indicators have a significant downfall due to the fact that they yield a low amount of fluorescence. In addition, the ultra-intensive laser (∼500 W/cm^2^) quickly brings side effects, such as heat damage to the tissue. These problems may be addressed by modifying these sensors to improve their brightness and photostability. The alternative method is to develop synthetic retinal analogs with strong absolute fluorescence (Sineshchekov et al., 2012; Herwig et al., 2017; Hontani et al., 2018). Also, due to the three-photon state mechanism described above, two-photon excitation tends to lose voltage sensitivity even with eFRET-based GEVIs (Maclaurin et al., 2013; Chamberland et al., 2017; Bando et al., 2019). Solving this problem would be a significant advance toward deeper-tissue voltage imaging.

One of the advantages of voltage imaging is to record neuronal activity from multiple neurons simultaneously. However, the time resolution and SNR of voltage imaging are still inferior compared to the patch-clamp recording. For practical use, voltage imaging needs to be able to measure subthreshold activity and decode absolute voltage from fluorescence changes. In fact, there have been several efforts to develop absolute voltage indicators based on microbial rhodopsins (Hou et al., 2014). Also, the fluorescent signal from population voltage imaging is correlated with local field potentials (Marshall et al., 2016; Bando et al., 2019; Piatkevich et al., 2019). Recently, implantable multi-electrode arrays (MEAs) are available for extracellular measurements of neural activity with high spatiotemporal resolution (Obien et al., 2014). Therefore, it is interesting to use MEAs for evaluating the performance of GEVIs.

The imaging apparatus and data processing play critical roles in voltage imaging. Membrane potential fluctuation occurs on a millisecond timescale. Thus, high-speed cameras with a large field of view are required to acquire images at a comparable frequency from multiple neurons. Also, an information processing system to handle big data is essential. For example, a size of 1-min voltage imaging by a sCMOS camera (512 pixels x 128 pixels, frame rate: 1 kHz) would be approximately 8 GB. Moreover, the signal of voltage imaging contains multiple waveforms. Data processing requires an accurate and coherent definition of APs, subthreshold activities, and background noise. Future directions need to focus on these aspects to improve the application of GEVIs with higher fidelity and reproducibility.

Despite these limitations, the development of GEVIs has allowed us to further investigate information dynamics and processes within neurons. Ultimately, voltage imaging will revolutionize the technology of imaging neural activity. It will make it possible to elucidate fundamental principles of how the brain functions, such as neuronal activity integration, information processing in micro-and long-range circuits, and the neuronal states.
